# Diagnostic Outcomes among Patients with Positive Multi-Cancer Early Detection Test Results

**DOI:** 10.1158/2767-9764.CRC-25-0723

**Published:** 2026-03-09

**Authors:** Elizabeth K. O’Donnell, Tia L. Kauffman, Sydney Asnis, Victoria A. Kelly, Ella Matthews, Matheos Kartsounis, Harita Dharaneeswaran, Jenna B. Beckwith, Ciola Bennett, Marjorie Marto, Giovanni Parmigiani, Timothy R. Rebbeck, Irene M. Ghobrial, Sapna Syngal, Catherine R. Marinac

**Affiliations:** 1 Dana-Farber Cancer Institute, Boston, Massachusetts.; 2 Harvard Medical School, Boston, Massachusetts.; 3 Harvard T.H. Chan School of Public Health, Boston, Massachusetts.; 4 Brigham and Women’s Hospital, Boston, Massachusetts.

## Abstract

Multi-cancer early detection (MCED) tests represent a significant technological advancement, allowing for the detection of multiple cancers through a single screening test. In December 2023, Dana-Farber Cancer Institute (DFCI) initiated an MCED clinical and research program to serve patients receiving positive MCED test results and further study MCED tests in clinical practice. Prospective case series reporting on patients with a signal detected by the Grail Galleri MCED test who presented for diagnostic evaluation at DFCI between December 2023 and January 2025. Primary outcomes were confirmation of cancer diagnosis, time to diagnostic resolution, and accuracy of the cancer signal origin (CSO) suggested. Of the 14 patients with a positive MCED test, 78.6% had a confirmed cancer diagnosis, whereas 21.4% were false positives. The median age was 62.5 years, with 64.3% male and 85.7% White, non-Hispanic. The median time from MCED test result to presentation at the DFCI MCED clinic was 28 days. The median time from MCED clinic presentation to diagnostic resolution was 20 days. Among confirmed cases, 54.5% were solid tumors and 45.5% were hematologic malignancies. More than 90% of detected malignancies lack current screening guidelines. Six cancers were diagnosed at stage I/II, and five at III/IV. Most patients with a cancer signal detected on a MCED test were true positives with a cancer consistent with the CSO reported. Patients had their MCED test results quickly adjudicated when presenting to a dedicated clinical program supporting a role for cancer diagnostic expertise in evaluating MCED tests.

**Significance:**

MCED tests are a new and evolving technology that may help detect cancer early on. Here, we report the experience of a dedicated cancer clinic in evaluating patients with a positive cancer signal from an MCED test.

## Introduction

Cancer remains a major public health challenge in the United States, with an estimated 2,041,910 new cancer cases and 618,120 cancer-related deaths projected in 2025 (1). Most cancers currently lack guidelines for recommended screening protocols (1). Although established cancer screening guidelines for breast, cervical, colorectal, and lung cancers have enabled early detection in some individuals (2–8), adherence to guideline-based screening is incomplete and varies widely across populations. As a result, the vast majority of cancers are usually detected incidentally or when a patient presents to their provider with symptoms, at which point the disease is often more advanced.

Recent technological advancements have facilitated the development of blood-based multi-cancer early detection (MCED) tests, which use a single test to detect multiple cancer types. These tests are designed to complement and not replace established screening paradigms, with the potential to broaden the range of cancers detected at an early stage. Importantly, current assays demonstrate high specificity (∼99%), reducing false positives and unnecessary downstream diagnostic evaluations (9). Such features have generated tremendous excitement for incorporating MCED tests as a cancer screening tool in routine clinical practice, with implications for both clinical care and population health.

Despite this promise, wider clinical adoption of MCED tests raises important challenges. Although MCED tests are already entering practice as laboratory-developed tests, many clinicians remain unfamiliar with them, and uncertainties persist regarding test interpretation, diagnostic follow-up, and integration into routine care. For example, in a 2023 cross-sectional survey of 88 primary care providers across the Mayo Clinic Enterprise, 61% reported either never having heard of MCED tests or having only minimal familiarity, whereas another 21% reported some knowledge without firsthand experience in ordering or interpreting results (10). In another survey of 351 primary care providers across a separate health system, respondents expressed general receptivity to MCED testing but also raised widespread concerns about the time and resources required to manage follow-up for patients with a signal-detected result (11). Together, these findings underscore the need for careful evaluation of MCED implementation in real-world settings to inform clinical management.

In 2023, the Dana-Farber Cancer Institute (DFCI) initiated a MCED clinic and research program to enhance understanding of emerging screening technologies and establish evidence-based approaches for managing individuals with positive MCED findings. In this report, we describe the workup and diagnostic outcomes of patients with signal-detected MCED tests evaluated at DFCI, providing early insights into the opportunities and challenges of managing patients with a signal-detected MCED testing in clinical practice.

## Materials and Methods

We conducted a prospective case series involving adults seen in the DFCI MCED clinic between December 1, 2023, and January 14, 2025, who presented with a prior positive cancer signal from the Grail Galleri MCED test. This blood-based assay detects multiple cancers by analyzing methylation patterns in cell-free DNA and provides up to two cancer signal origins (CSO; refs. 9, 12–14). Patients presenting to the DFCI MCED clinic with a positive MCED test result were evaluated exclusively by a single medical oncologist; the clinical team also included a nurse and physician assistant. The study was approved by the Dana-Farber/Harvard Cancer Center Institutional Review Board [#: 25-190, 22-200 (NCT05463796)].

Data collection was systematically conducted using standardized forms, tracking patient information as part of routine clinical practice. Data extracted from the electronic medical record included demographics (age, sex, race, and ethnicity), lifestyle factors (smoking history, alcohol use, and body mass index), medical history (personal cancer screening history and family and personal history of cancer), and clinical workup details, such as MCED test dates, CSO results, diagnostic procedures, diagnostic resolution date, retest information, and cancer diagnosis/stage, if applicable.

The clinician also collected information on referral pathways, including whether the individual was from in-state (Massachusetts) or out-of-state, and source of referrals. Details on diagnostic tests, including types of biopsies, advanced imaging, and blood and laboratory tests, were abstracted after the procedures were completed. Diagnostic resolution was determined by the MCED clinic physician and established through pathologic and radiographic confirmation. Descriptive statistics were used to summarize patient characteristics and clinical outcomes. We calculated the time from a positive MCED signal to the initial DFCI appointment and to diagnostic resolution using exact dates.

## Results

Fourteen patients with a positive cancer signal from the Grail Galleri MCED test were evaluated at the DFCI MCED program between December 1, 2023, and January 14, 2025. The median age was 62.5 years (range, 54.9–81.4 years), with 64.3% (9/14) male and 85.7% (12/14) self-identifying as White, non-Hispanic (Table 1). A personal cancer history was noted in 28.6% (4/14) of patients, with age of diagnosis ranging from 41 to 55 years old; prior cancers included prostate (*N* = 1), testicular (*N* = 1), and thyroid cancers (*N* = 2).

**Table 1.** Characteristics of adults with cancer signal detected by Grail Galleri Test at DFCI MCED^a^ Program (*n* = 14).

​	*n* (%)	Cancer diagnosed *n* (%)
Overall	14 (100%)	11 (78.6%)
Median age (range) (years)	62.5 (54.9–81.4)	60.6 (54.9–81.4)
Sex	​	​
Female	5 (35.7%)	4 (28.6%)
Male	9 (64.3%)	7 (50%)
Race/ethnicity	​	​
White	12 (85.7%)	10 (71.4%)
Non-White	2 (14.3%)	1 (7.1%)
Smoking	​	​
No	8 (57.1%)	6 (42.9%)
Yes, past history	6 (42.9%)	5 (35.7%)
BMI^b^ category	​	​
Underweight	1 (7.1%)	1 (7.1%)
Normal	4 (28.6%)	3 (21.4%)
Overweight	6 (42.9%)	4 (28.6%)
Obese	3 (21.4%)	3 (21.4%)
Alcohol intake	​	​
None	3 (21.4%)	1 (7.1%)
1–6 drinks/week	6 (42.9%)	5 (35.7%)
1+ drink per day	5 (35.7%)	5 (35.7%)
Referrals	​	​
In-state (Massachusetts)	6 (42.9%)	5 (35.7%)
Out-of-state^c^	8 (57.1%)	6 (42.9%)
Personal history of cancer	4 (28.6%)	3 (21.4%)
Family history of cancer	14 (100%)	11 (78.6%)

Among 14 adults with a positive MCED signal.

a DFCI MCED.

b Body mass index.

c Out-of-state incudes New York, New Hampshire, Maine, Florida, California, and Pennsylvania.

The majority of participants [92.8% (13/14)] presenting with a positive MCED test in our clinical program were up to date with guideline-based standard-of-care (SOC) screening. Ninety-three percent (13/14) had previously undergone colon cancer screening. All five females were up to date with their cervical cancer screenings, and 80% (4/5) were up to date with their breast cancer screenings. Lung cancer screening was conducted for one former smoker, and skin cancer screening was reported in 57.1% (8/14). Among the nine males, 77.8% (7/9) were previously screened for prostate cancer, with four screened within the past year.

Participants received their initial MCED tests through various sources: 35.7% (5/14) through their employer, 28.6% (4/14) through an employer-sponsored or insurer-affiliated research or pilot program, 21.4% (3/14) from the MCED company website, 7.1% (1/14) through a primary care provider, and 7.1% (1/14) from a specialist healthcare provider (Table 2). Patients with positive MCED tests were from both in-state and out-of-state sources, with 57.1% (8/14) being from outside of Massachusetts. Most patients self-referred to the DFCI MCED clinic [64.3% (9/14)] for their diagnostic evaluation, 21.4% (3/14) were referred by other healthcare providers, and 14.3% (2/14) were referred by their employer.

**Table 2.** Source of baseline MCED tests and referral patterns.

Source of baseline MCED Tests	*n* (%)
Employer	5 (35.7%)
Insurance company	4 (28.6%)
MCED company website	3 (21.4%)
Primary care provider	1 (7.1%)
Specialist healthcare provider	1 (7.1%)

Referral Source for DFCI MCED Program	*n* (%)
Self-referred	9 (64.3%)
Primary care provider	3 (21.4%)
Employer	2 (14.3%)

The primary and secondary CSOs from the MCED test and the diagnostic evaluations performed are detailed in Supplementary Table S1. All patients underwent advanced imaging as part of their diagnostic workup. To achieve diagnostic resolution, true positive cases required a median of three diagnostic tests or procedures (range, 2–7), whereas false positives required a median of five tests (range, 4–6). Following diagnostic evaluation, 78.6% (11/14) had confirmed cancer diagnoses, whereas 21.4% (3/14) were deemed false positives. Among confirmed cases, 54.5% (6/11) were solid tumors, including triple-negative breast, testicular, liver, cholangiocarcinoma, lung (nonsmoker), and tonsillar cancers. The remaining 45.5% (5/11) were hematologic malignancies, comprising four lymphomas and one patient with high-risk smoldering myeloma. Notably, more than 90% (10/11) of the detected malignancies lack current screening guidelines. Six cancers (54.5%) were diagnosed at stage I/II, and five (45.5%) at stage III/IV. The first or second CSO was accurate in more than 90% (10/11) of cases for those confirmed to have cancer. No biopsies were conducted in false positive cases. Based on this experience, we provide our general approach for performing a clinical evaluation of a patient with a cancer signal-detected test result in Supplementary Table S2.

The referral and diagnostic timelines for the 14 patients presenting with a cancer signal–detected MCED test are presented in the Supplementary Appendix. The median time from receiving the MCED test result to presentation at DFCI was 28 days (range, 6–368 days). Several patients initiated diagnostic evaluations locally, either by the ordering clinician or another primary care provider involved in their care. Details of the diagnostic evaluations are included in Supplementary Appendix. Among true positives, the median interval from the MCED test to the initial DFCI visit was 25 days (range, 6–368 days). The outlier at 368 days reflected a unique case: this patient had clear evidence of disease identified by the first CSO on day 49 but did not present to the DFCI MCED clinic until day 368, following referral for evaluation of a second CSO (see P13 timeline). For false positives, the median time from receiving the MCED test result to presentation at DFCI was 48 days (range, 7–75 days). Diagnostic evaluation took a median of 20 days, in which true positive cases resolved more quickly (median of 15 days) compared with false positives (median of 98 days). For true positive cases, the time from the initial DFCI clinic visit to diagnostic resolution ranged from 5 to 78 days (median 15 days). In contrast, false positive cases took longer to resolve, with times from the initial DFCI clinic visit to resolution ranging from 92 to 104 days (median 98 days). All three false positive cases underwent repeat MCED testing after a median of 118 days, with no cancer signal detected upon retesting. One additional MCED retest occurred to further adjudicate the second CSO that was originally detected, because initial imaging for the second CSO was inconclusive and was negative upon retest.

Overall, there were no issues with prior authorizations or insurance denials for the diagnostic evaluations. There were no adverse events or complications from the diagnostic evaluations reported. Nine of 11 true positives received treatment at our cancer center.

## Discussion

This study reports on the clinical pathways and diagnostic outcomes of individuals with a signal-detected MCED test presenting to a specialized diagnostic program, demonstrating that 78.6% of those who presented for clinical evaluation were ultimately diagnosed with cancer. The test accurately identified the first or second CSO in 90% of cases, indicating its potential value in guiding diagnostic decisions. Notably, 60% of detected cancers were at stage I or II, and 90% of malignancies identified had no current screening guidelines; individuals presenting to our clinic were also largely compliant with SOC screenings. Together, these findings support the notion that MCED tests may have the potential to detect cancers at an early stage as well as broaden the range of cancers that can be detected through screening (2–7).

The proportion of patients evaluated with a signal-detected MCED test who were confirmed to have cancer in our sample was notably higher than reported in prior prospective interventional studies (15). We hypothesized that patients who sought evaluation at a specialized diagnostic program may have had additional risk factors or clinical concerns that heightened their suspicion for cancer. However, recent data from a pragmatic trial offering MCED screening to individuals in a tertiary referral center also demonstrated a similarly high positive predictive value [PPV; 73.3% (11/15); ref. 16]. In that cohort of 2,244 asymptomatic patients who underwent MCED testing over 18 months, 17 individuals tested positive for a cancer signal. Of those, 15 completed further diagnostic evaluations, and cancer was confirmed in 11 cases (73.3%). These consistently high PPVs observed in our study and in the executive health clinic described above raise important questions about factors that contribute to the discordance with other published prospective data. Possible explanations include inherent selection bias among individuals who pursue MCED testing outside of research protocols, many of whom pay out-of-pocket (often >$900) and may have a higher underlying risk of cancer due to age, family history, environmental exposures, or prior medical conditions. Additionally, the use of coordinated, multidisciplinary diagnostic pathways in specialized programs may enhance the accuracy of follow-up evaluations and the ability to correctly resolve true positive cases. Together, these findings highlight the need for further research to clarify the factors driving the variability in PPV across diverse clinical contexts and refine diagnostic algorithms, ensuring that patients with positive MCED results are evaluated by providers with expertise in both the test characteristics and the appropriate diagnostic strategies needed to adjudicate results.

One of the key challenges of MCED testing is managing patients who receive a positive cancer signal but have no malignancy detected after a thorough diagnostic evaluation. In addition to having to undergo a long diagnostic process, this can result in heightened and protracted anxiety. One evidence-based tool our clinic relied on in this subset of patients was the free retest offered by GRAIL. Specifically, in data previously reported, Westgate and colleagues evaluated the utility of this retest and found that, among patients with a negative diagnostic workup following an initial positive test, 69% had no cancer signal detected upon retesting at a median interval of 5.4 months. Notably, none of the patients who tested negative on retest had developed incident cancers at the time of analysis, whereas 29% of patients in that sample with persistently positive results on retesting were later diagnosed with cancer, suggesting that those with persistently positive signals require close survellience (17). In our study sample, individuals with a positive MCED test and a negative diagnostic evaluation underwent repeat testing at a median of 118 days. Of the three patients with negative diagnostic workups that were retested, all received “no signal detected” results on their retests. The disappearance of a cancer signal, combined with negative clinical evaluations, concluded the diagnostic evaluation for these patients; these individuals were considered to be false positive cases and were counseled to continue SOC, guideline-recommended cancer screenings, with clear communication that MCED testing is not a replacement for established screening methods.

A central challenge to the effective integration of MCED tests into clinical practice is ensuring timely access to providers who can interpret and act on results. Currently, no MCED test has received FDA approval; however, they are available to patients either through their healthcare providers or via direct-to-consumer channels. In our clinic, 12 of the 14 patients (86%) reported obtaining their test either as an employee benefit or directly from the manufacturer’s website, whereas only two patients received testing through their healthcare providers. For those accessing tests outside of their usual care, the responsibility of identifying a clinician to interpret and manage a positive result often fell to the patient. Indeed, the most frequently cited issue among patients in our sample was finding a provider who was both familiar with the test and comfortable conducting the necessary diagnostic evaluation, thus contributing to the high percentage of patients presenting to the DFCI MCED clinic from out of state. Once seen by a qualified provider in our program, however, the results were adjudicated quickly. These findings underscore the gap between tests ordered direct-to-consumer and connecting with a provider familiar and willing to conduct the evaluation of the positive results.

Importantly, challenges are not limited to access; inappropriate follow-up by providers can also place patients at risk. We observed several instances in which patients with positive MCED test results underwent inappropriate diagnostic evaluations by the same providers who ordered the MCED test or by primary care providers asked to evaluate their own patients when they had not been the ordering provider. In four of the 14 cases, the patients either received inappropriate studies for the evaluation of the cancer suggested or were falsely reassured by their providers that they did not need further testing. In each of these cases, the individuals were ultimately diagnosed with a cancer consistent with the signal of origin suggested by the MCED test. These evaluations raise concerns about substandard care and highlight the urgent need to establish standardized protocols for MCED test follow-up and ensure evaluation by qualified providers which could include a medical oncologist, internist, or advanced practice provider appropriately trained in the properties of the MCED test administered. Such safeguards will be essential to protect patients, particularly as additional MCED tests enter the commercial market, and will play a critical role in maximizing benefit while minimizing potential harm.

Finally, there are limited data that inform the frequency at which MCED tests should be used, including those with false positive tests. The only published prospective data to date was for one-time only screening. In the PATHFINDER study, patients received the Galleri test and were followed for outcomes for 12 months (15). The ongoing NHS Galleri study is a randomized trial of the Galleri test versus SOC cancer screening, in which patients received the Galleri test a total of 3 times, each 12 months apart. The goal of that study is to determine whether screening with Galleri can reduce the likelihood of a late-stage cancer diagnosis in asymptomatic patients. The primary outcome is the number of stage III to IV cancers diagnosed. We hope that the NHS study will provide prospective, evidence-based information to share with our patients about the cadence of MCED testing through the number of cancers detected in individuals receiving serial testing.

In summary, our clinical experience of adjudicating tests within a specialized MCED program highlights the potential of these tests to complement and expand established screening strategies, particularly for cancers in which there is no routine screening guideline. Most patients who presented with a cancer signal detected on an MCED test were ultimately diagnosed with a cancer consistent with the detected signal of origin. Although some patients initially faced delays in securing a provider with experience managing MCED test results, our clinical program effectively facilitated rapid adjudication, highlighting the importance of specialized diagnostic expertise and dedicated clinical pathways in enhancing the efficiency of cancer detection and diagnosis.

## Supplementary Material

Supplementary Appendix Supplementary Appendix shows the referral and diagnostic timelines for the 14 patients presenting with a cancer signal-detected MCED test.

Supplementary Table 1 Supplementary Table 1 shows the primary and secondary CSOs from the MCED test and the diagnostic evaluations performed.

Supplementary Table 2 Supplementary Table 2 shows our general approach for performing a clinical evaluation of a patient with a cancer signal-detected test result.

## Data Availability

Data collected for this analysis and related documents will be made available to others upon reasonably justified requests. Requests for data sharing need to be written and addressed to the corresponding author at the following e-mail address: Elizabeth_odonnell@dfci.harvard.edu. The sponsor of the trial, the Massachusetts General Hospital, via the corresponding author, reserves the right to decide whether or not to share the data. Access will be provided after a proposal has been approved by an independent review committee established for this purpose and after receipt of a signed data sharing agreement. Additionally, all data will be disclosed consistent with all applicable federal statutes and regulations for the protection of human subjects, including, if applicable, the *Standards for Privacy of Individually Identifiable Health Information* set forth in 45 C.F.R. Part 164.
