# Supplementary figures and images for: Diagnostic Outcomes among Patients with Positive Multi-Cancer Early Detection Test Results
Source: Cancer Res Commun. 2026 Mar 9;6(3):511–5. doi: 10.1158/2767-9764.CRC-25-0723 (PMC13012066; doi:10.1158/2767-9764.CRC-25-0723)

## Slide 1
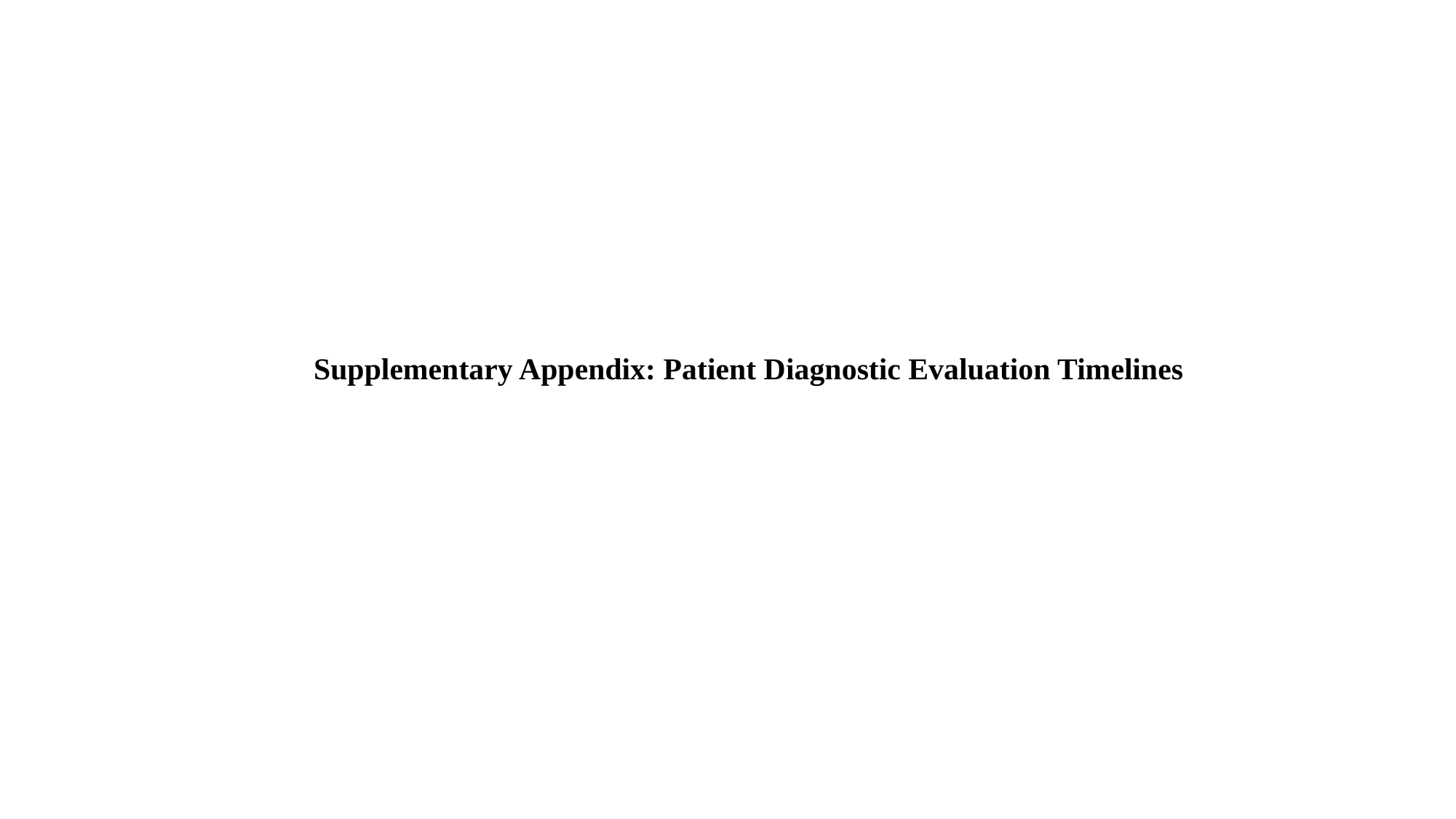

## Slide 2
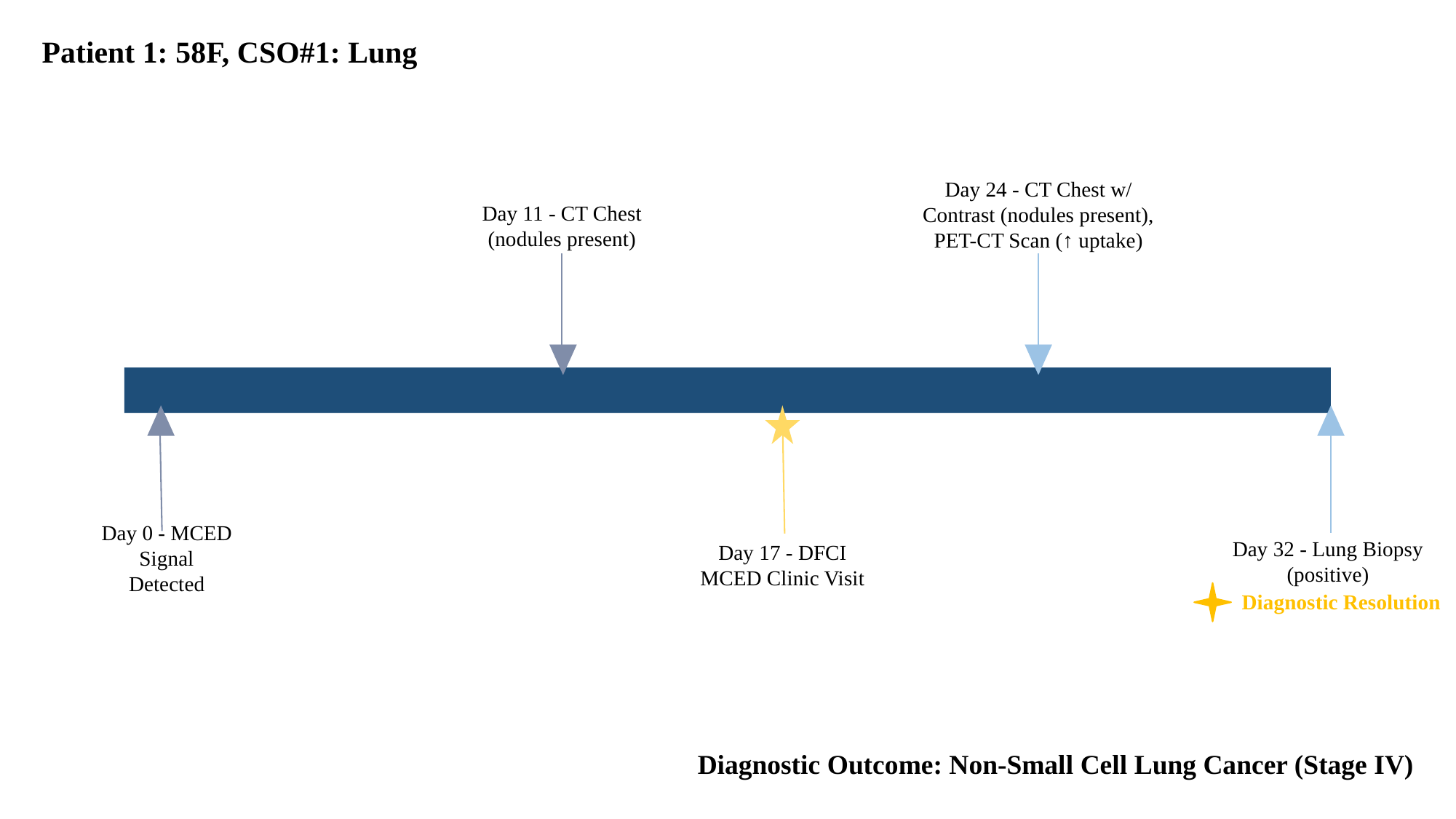

## Slide 3
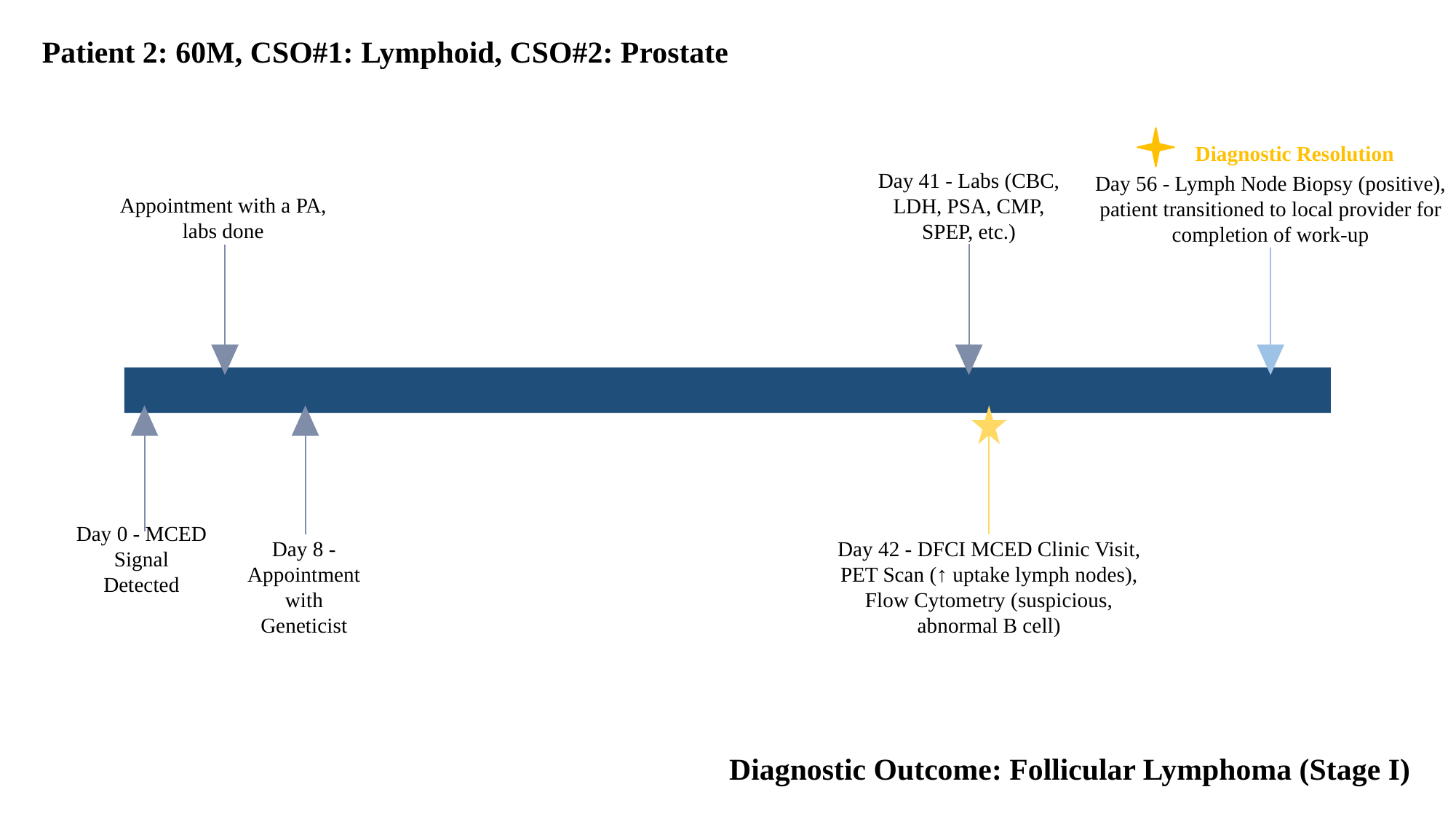

## Slide 4
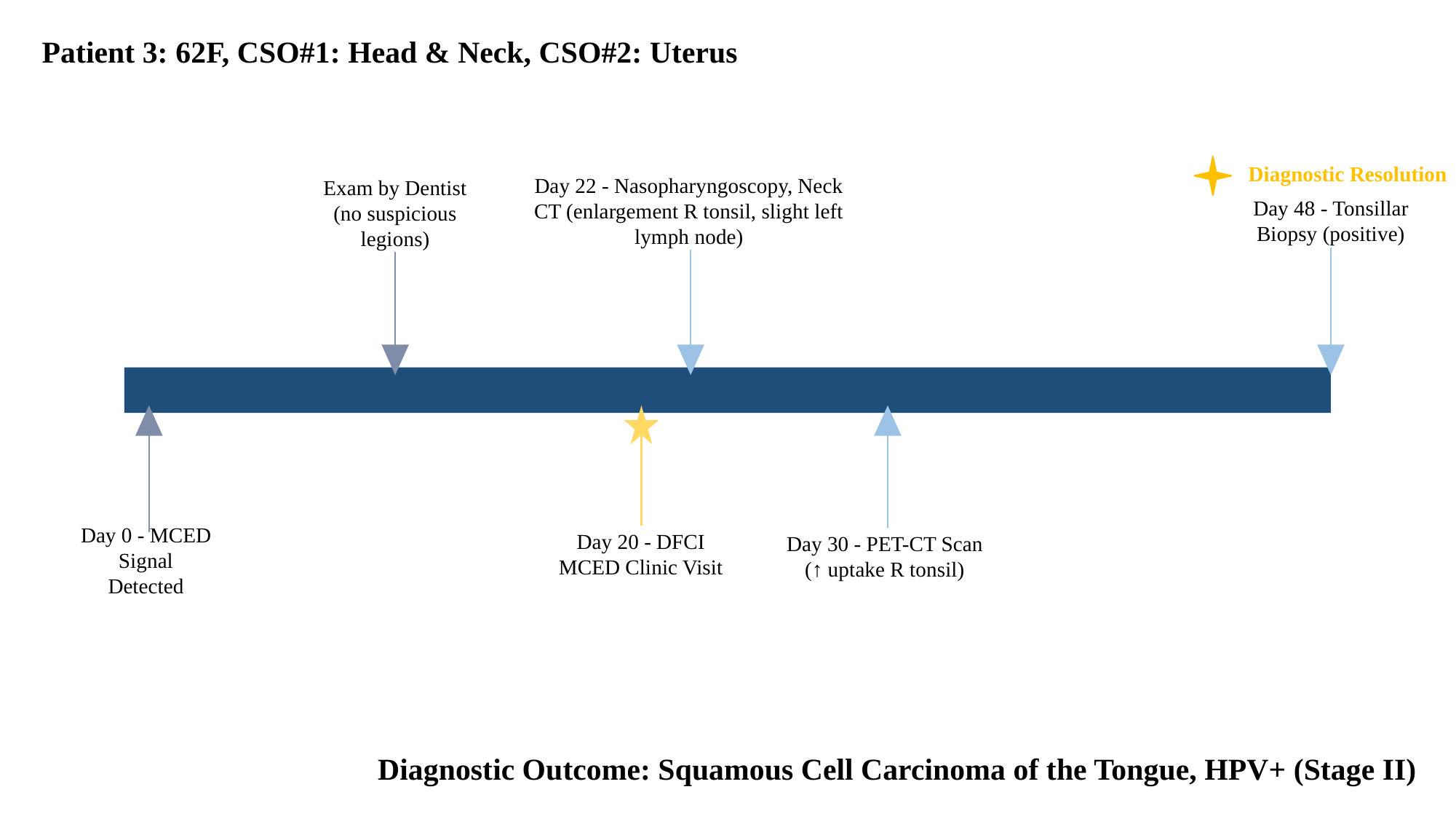

## Slide 5
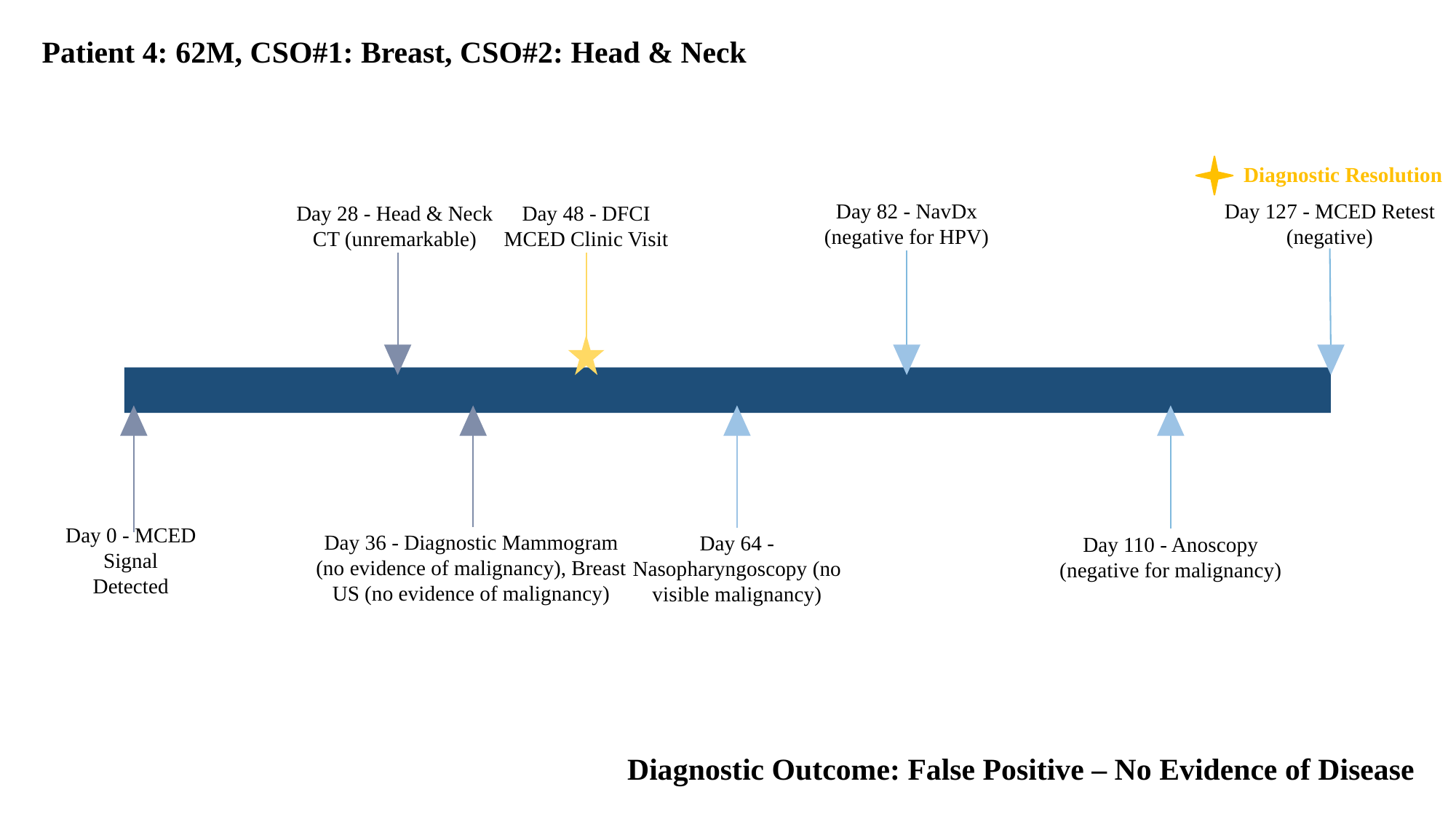

## Slide 6
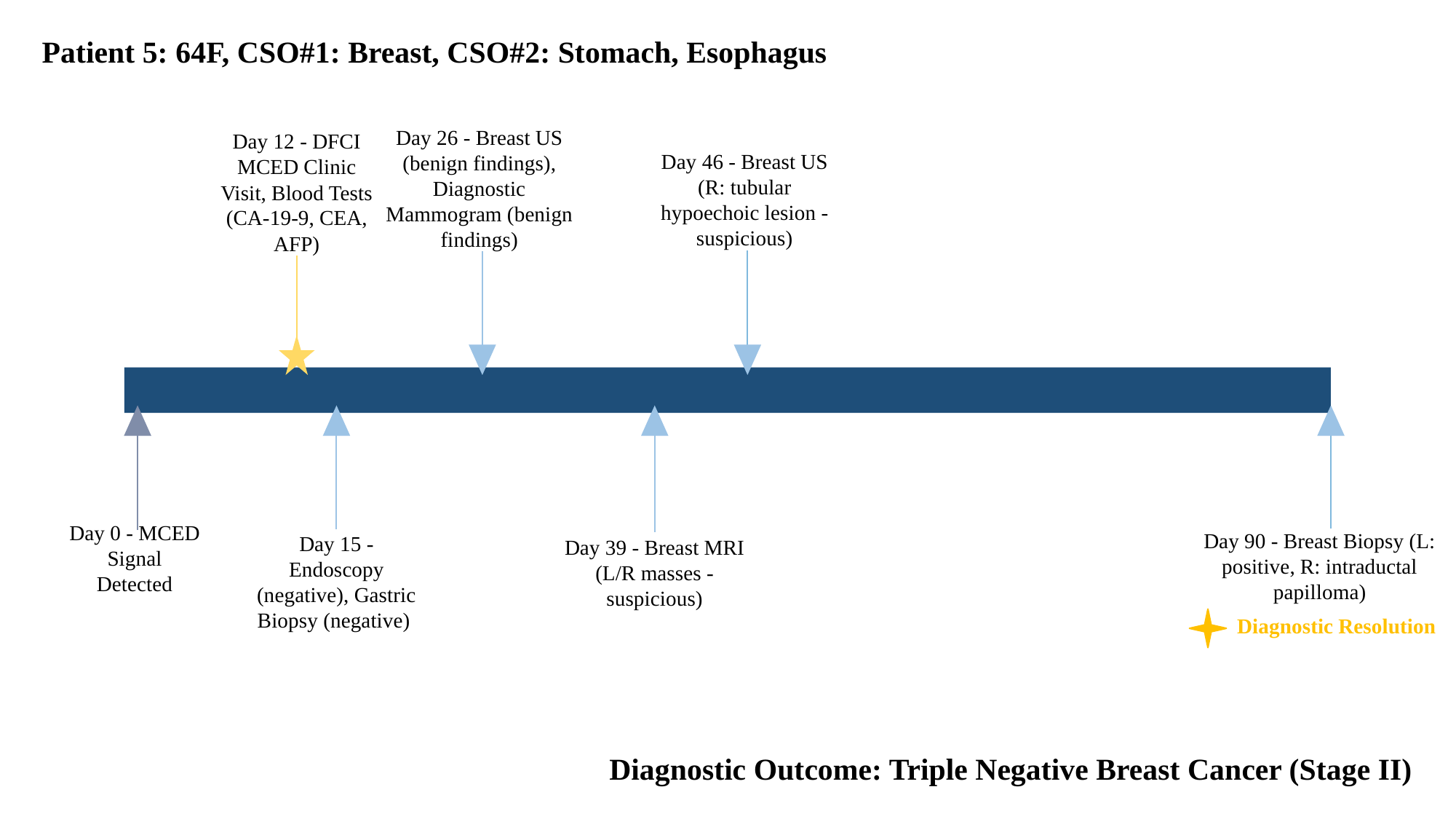

## Slide 7
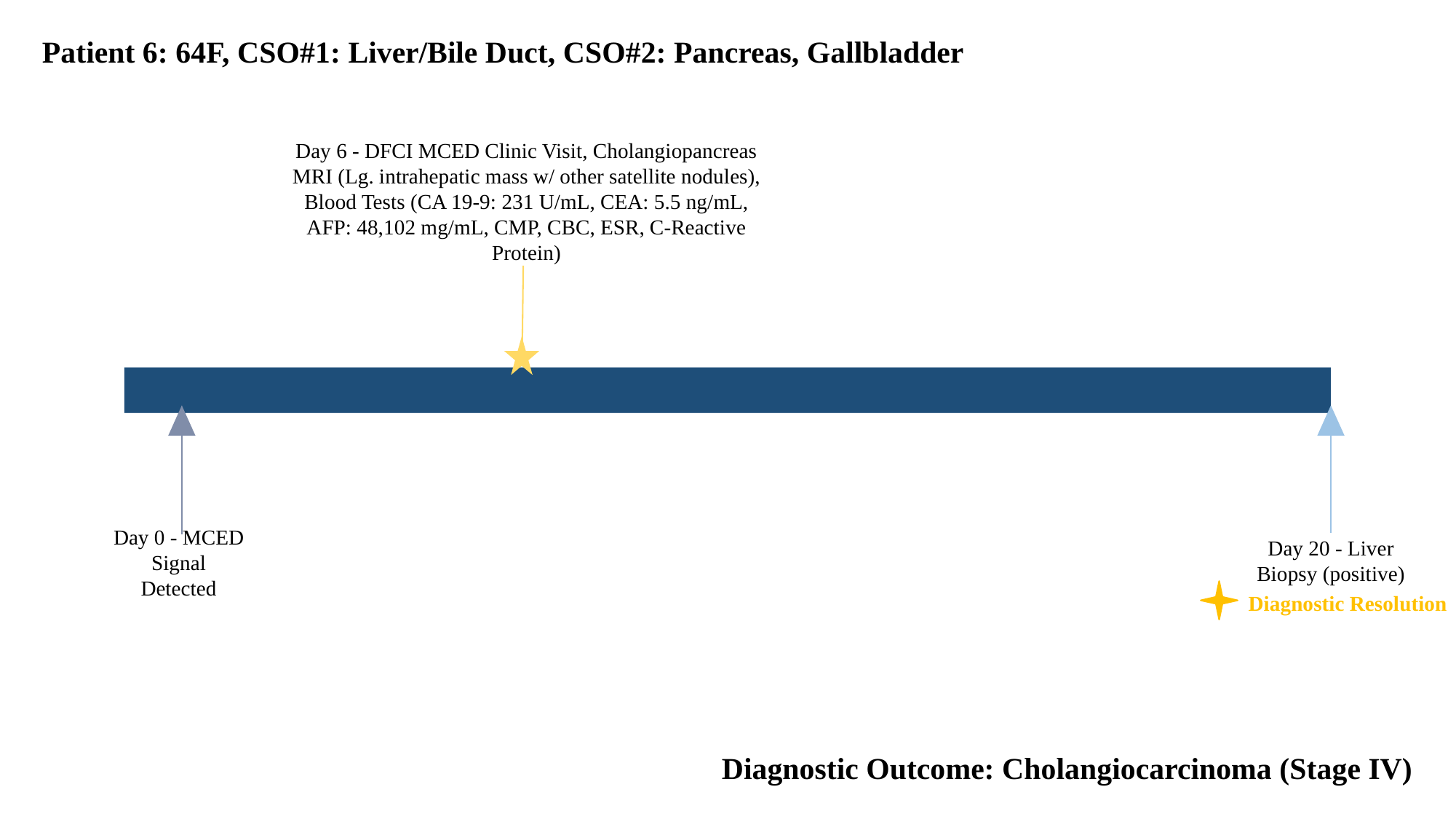

## Slide 8
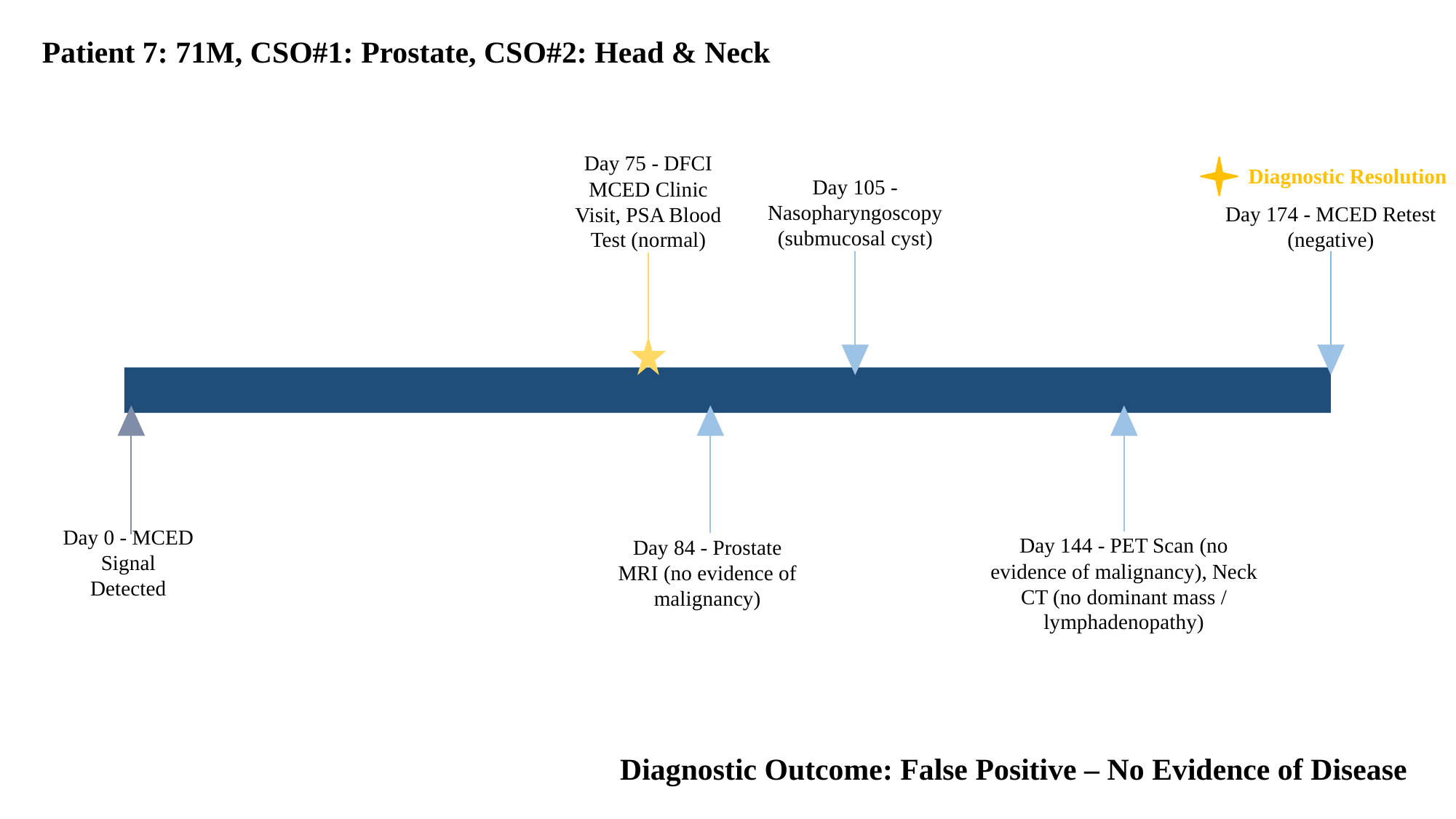

## Slide 9
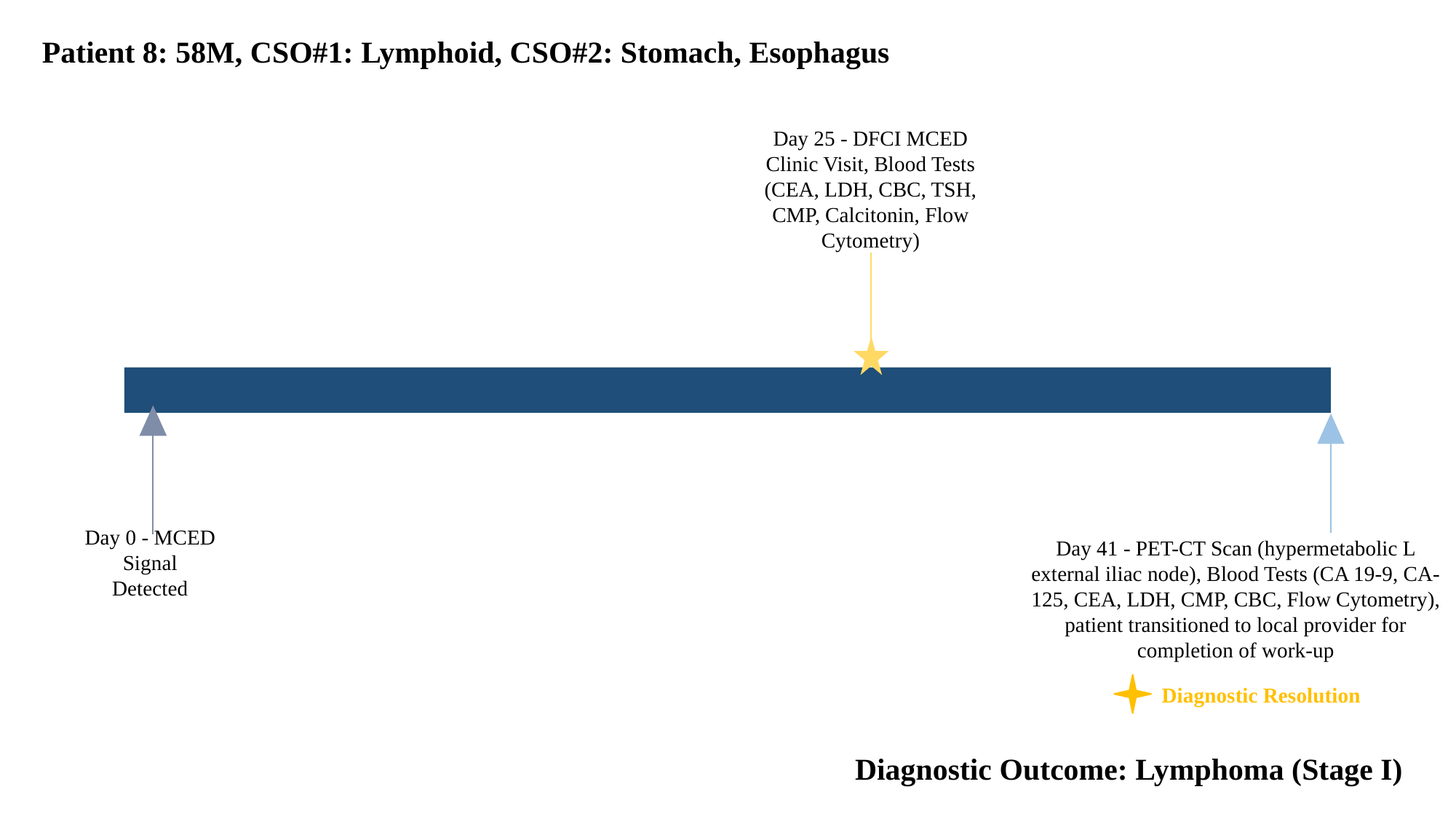

## Slide 10
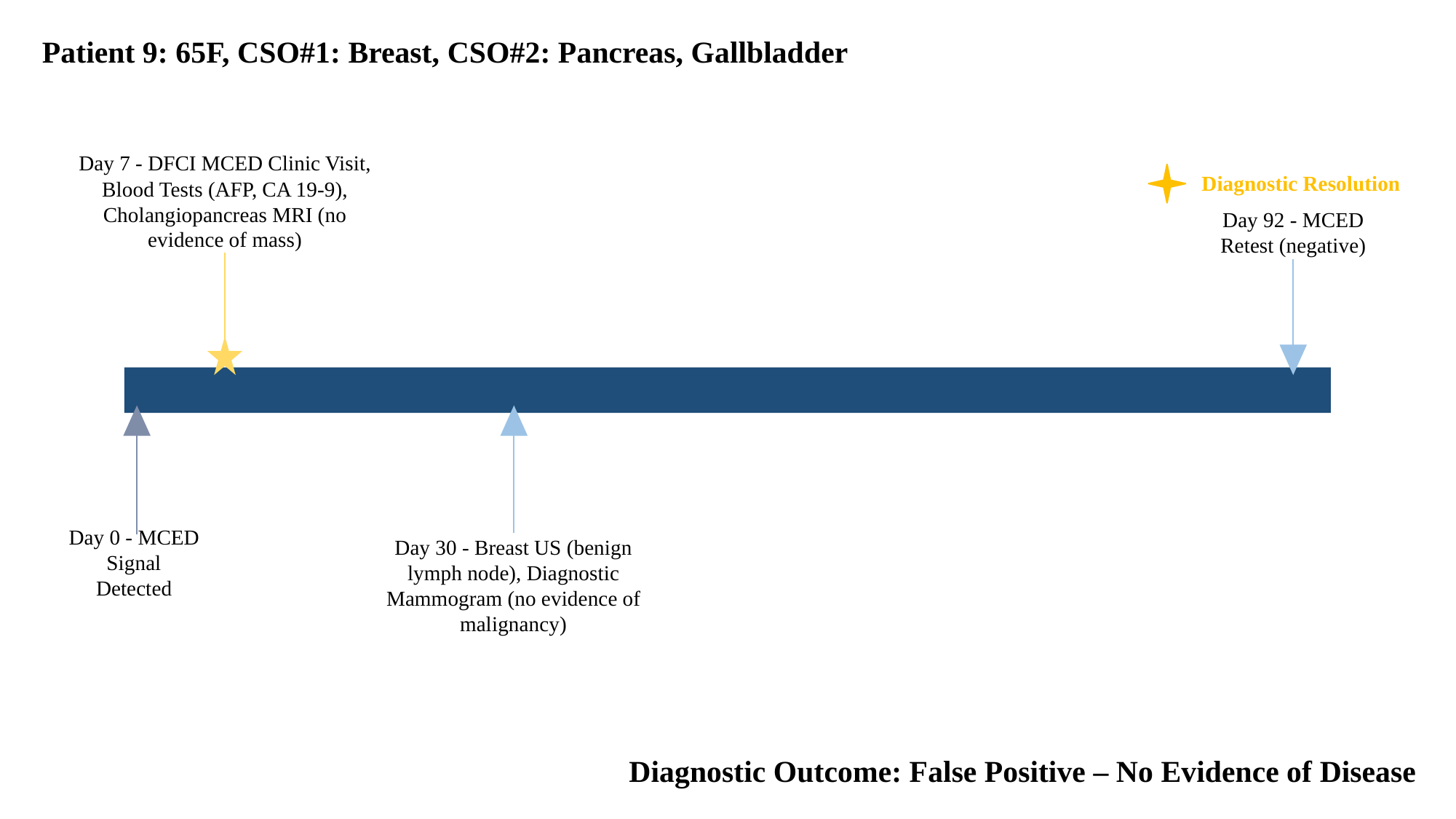

## Slide 11
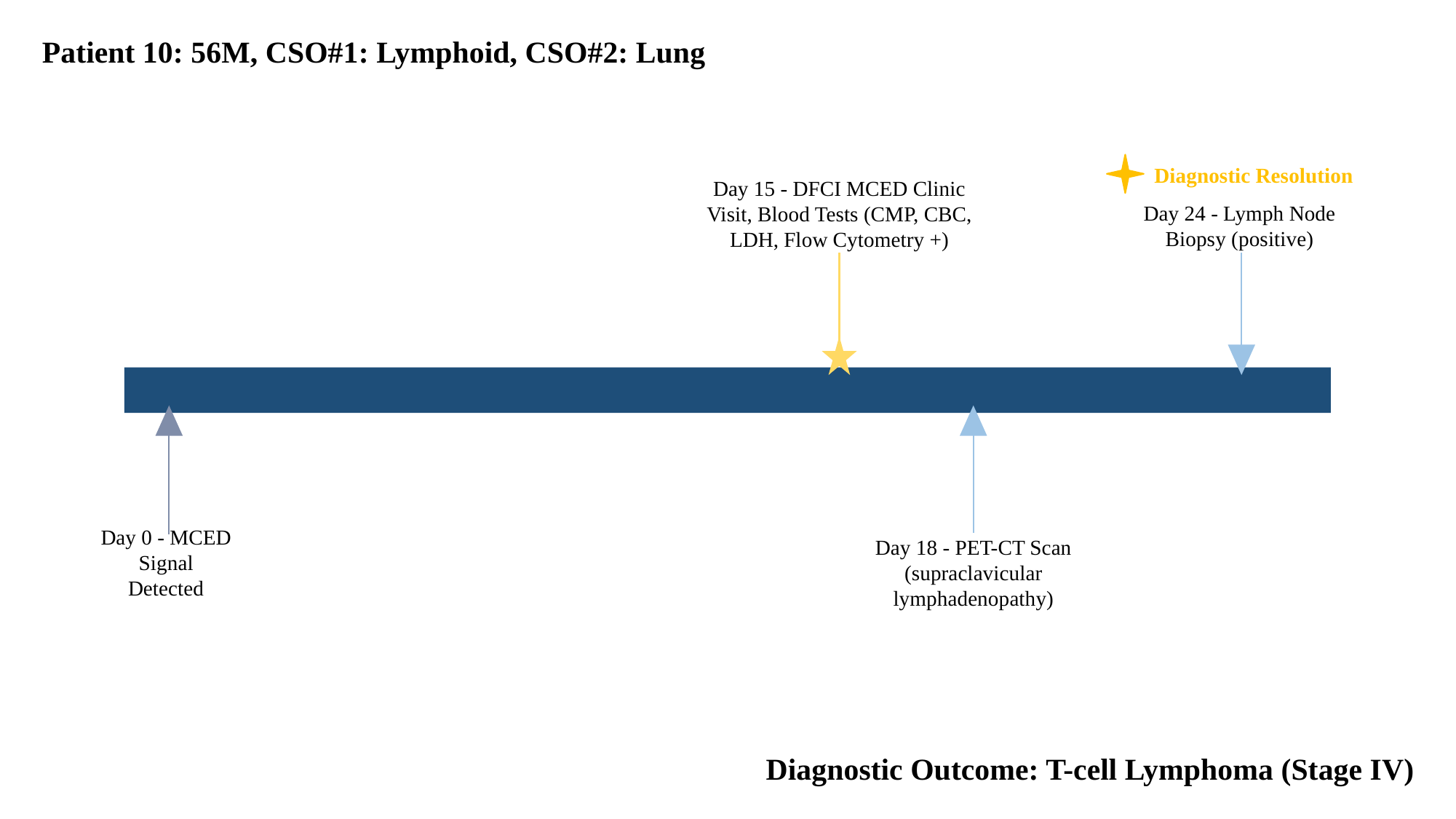

## Slide 12
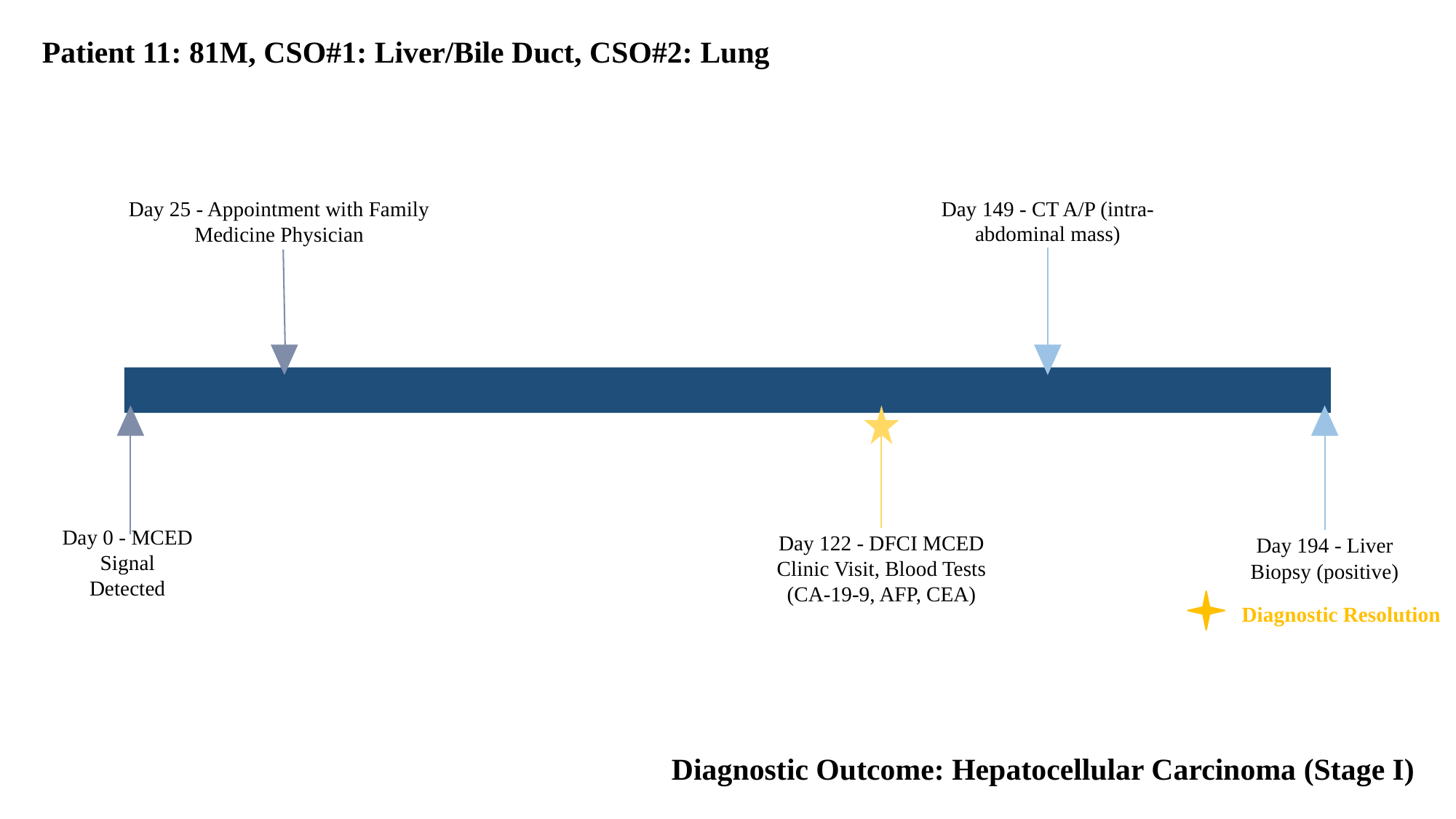

## Slide 13
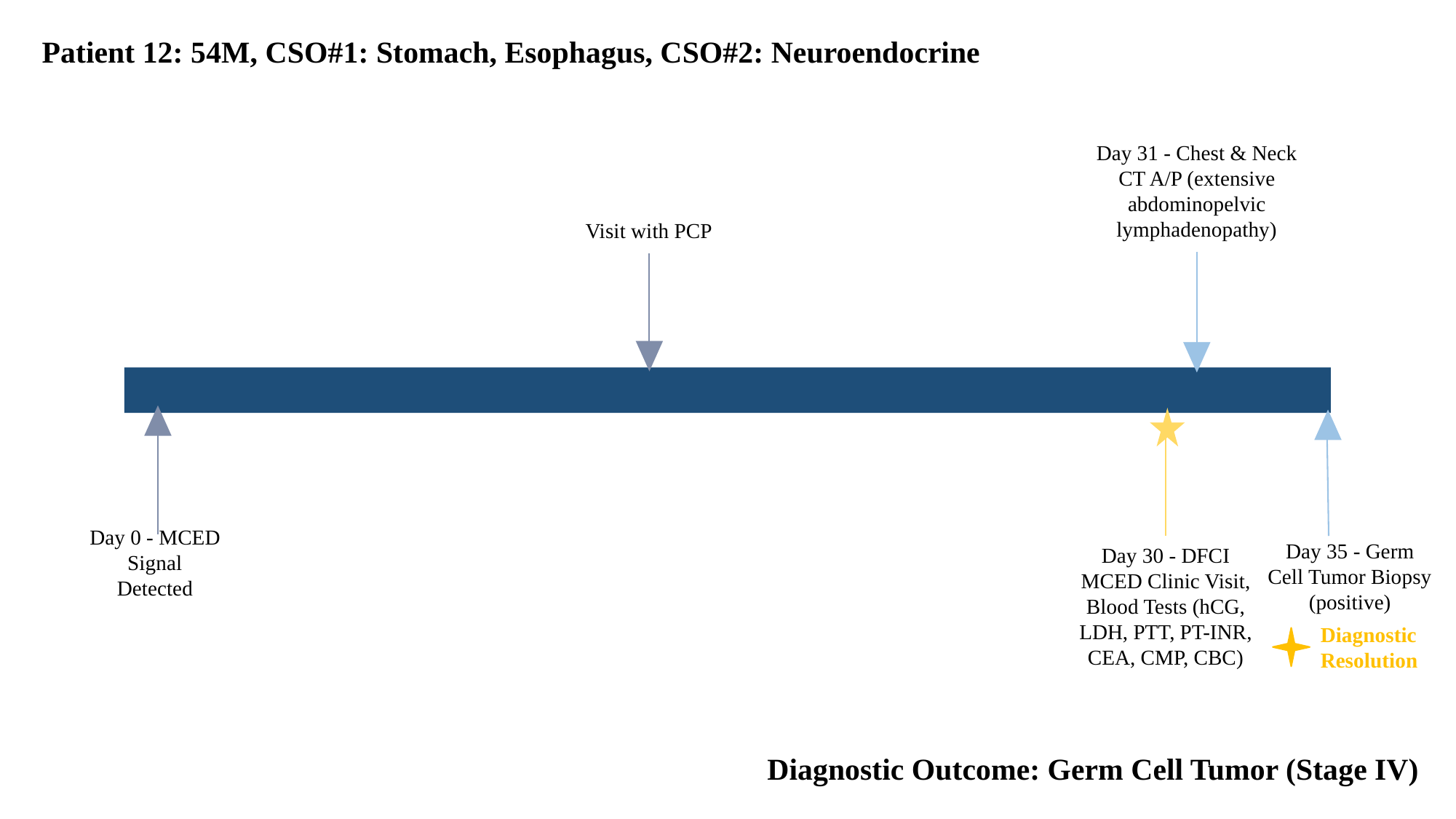

## Slide 14
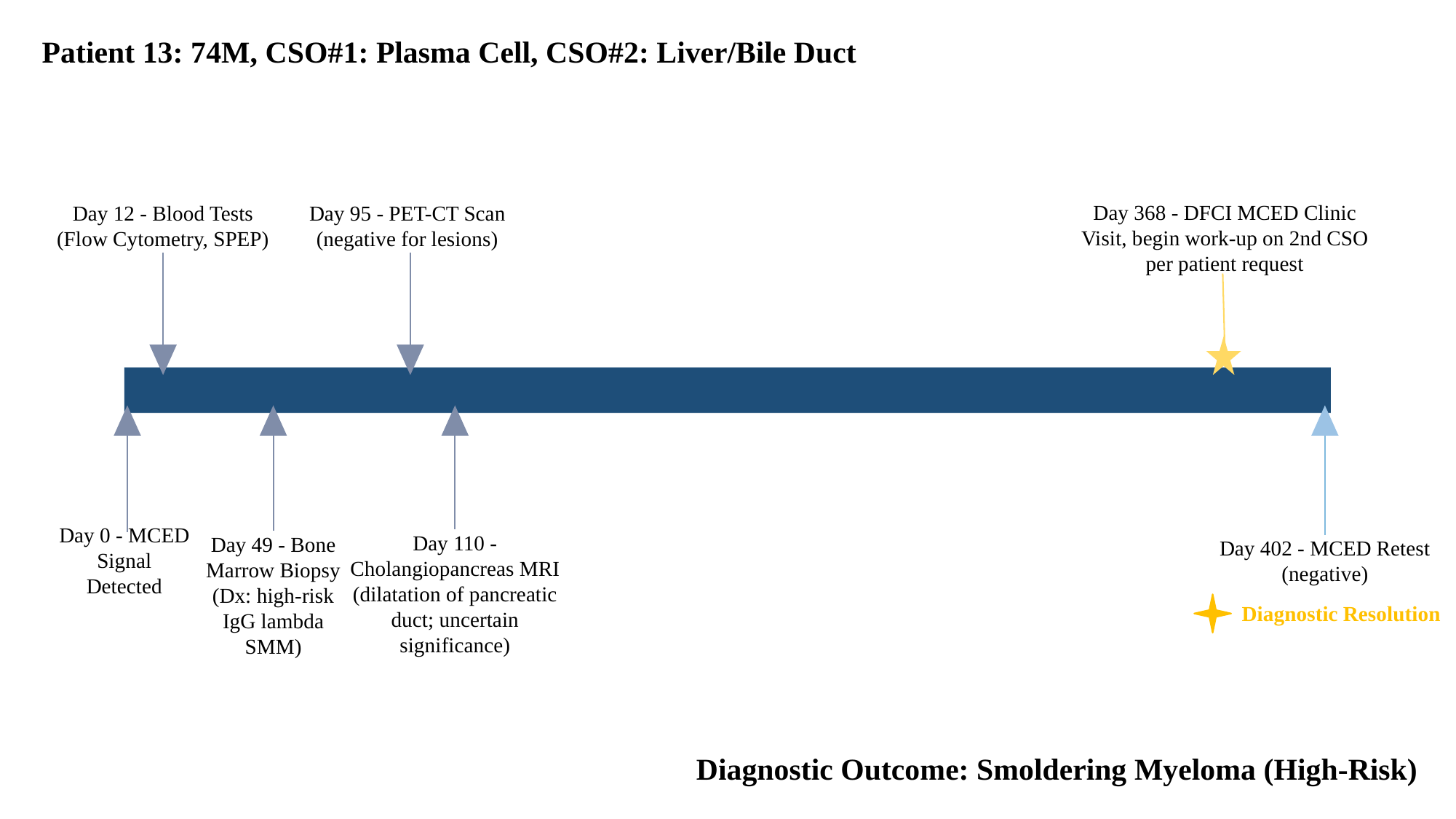

## Slide 15
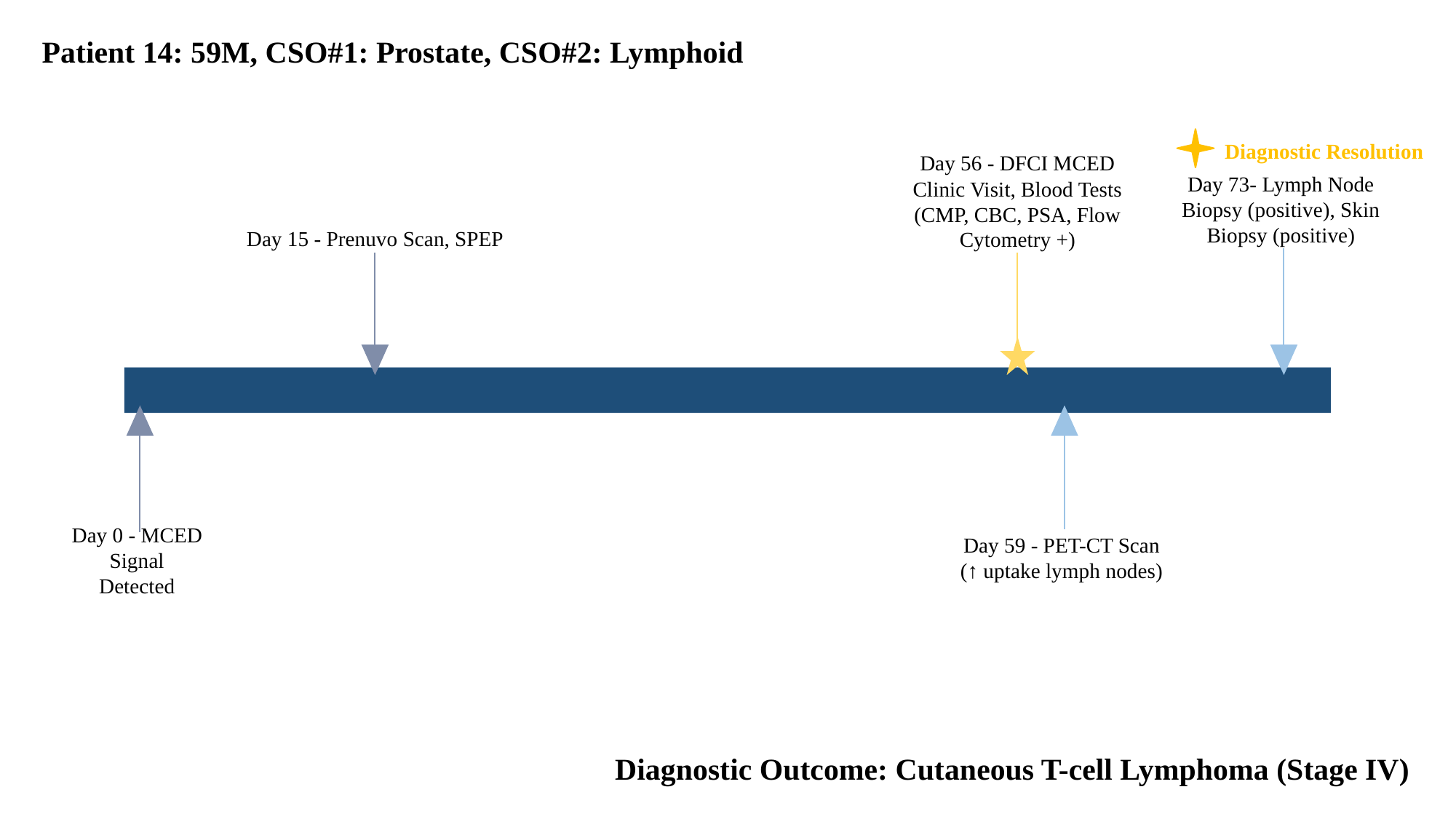

Supplement: Supplementary Appendix — shows the referral and diagnostic timelines for the 14 patients presenting with a cancer signal-detected MCED test. [file crc-25-0723_supplementary_appendix_suppsa.pptx]
