# Supplementary material for: Diagnostic Outcomes among Patients with Positive Multi-Cancer Early Detection Test Results
Source: Cancer Res Commun. 2026 Mar 9;6(3):511–5. doi: 10.1158/2767-9764.CRC-25-0723 (PMC13012066; doi:10.1158/2767-9764.CRC-25-0723)
Supplement: Supplementary Table 1 — shows the primary and secondary CSOs from the MCED test and the diagnostic evaluations performed. [file crc-25-0723_supplementary_table_1_suppst1.docx]

**Supplementary Table 1.** Cancer Signal Origin, Diagnostic Evaluations, and Clinical Diagnoses

| **Patient** | **Cancer Signal Origin** | **Work-up** | **Outcome** | **Stage** |
| --- | --- | --- | --- | --- |
| 58F | 1. Lung | - Low-dose chest CT - Chest CT with contrast - PET scan - Bronchoscopy with biopsy | Non-small cell lung cancer | IV |
| 64M* | 1. Lymphoid 2. Prostate | - Flow cytometry - PET scan - Lymph node biopsy | Follicular Lymphoma | I |
| 62F | 1. Head & Neck 2. Uterus | - Nasopharyngoscopy - CT Neck - PET CT - Biopsy | Squamous cell carcinoma of the tonsil, HPV positive | II |
| 62M | 1. Breast 2. Head & Neck | - CT head & neck - Nasopharyngoscopy - Breast ultrasound - Mammogram - Nasopharyngoscopy - Anoscopy - Galleri re-test (Negative) | No evidence of malignancy | N/A |
| 64F | 1. Breast 2. Stomach, Esophagus | - Blood tests: CEA, CA19-9 - Endoscopy with gastric biopsy - Breast ultrasound - bilateral - Diagnostic mammogram - Breast MRI - Breast ultrasound - right - Biopsy breast | Triple negative breast cancer | II |
| 64F | 1. Liver/Bile Duct 2. Pancreas, Gallbladder | - Blood tests: AFP, CA19-9, CEA - MRI cholangiopancreas - Biopsy liver | Cholangiocarcinoma | IV |
| 71M | 1. Prostate 2. Head & Neck | - Blood test: PSA - MRI prostate - Nasopharyngoscopy - CT neck - PET CT - Galleri re-test: Negative | No evidence of malignancy | N/A |
| 58M* | 1. Lymphoid 2. Stomach, Esophagus | - Blood tests: CA19-9, CEA, LDH, flow cytometry - PET scan | Lymphoma | I |
| 65F | 1. Breast 2. Pancreas, Gallbladder | - Blood tests: AFP, CA19-9 - MRI cholangiopancreas - Breast ultrasound - Diagnostic mammogram - Galleri re-test: Negative | No evidence of malignancy | N/A |
| 56M* | 1. Lymphoid 2. Lung | - Blood tests: CBC, LDH, flow cytometry - PET CT - Biopsy lymph node | Nodal T Follicular Helper Cell Lymphoma | IV |
| 81M | 1. Liver/Bile Duct 2. Lung | - Blood tests: AFP, CA19-9, CEA - CT abdomen/pelvis - Biopsy liver | Hepatocellular Carcinoma | I |
| 54M | 1. Stomach, Esophagus 2. Neuroendocrine | - Blood tests: LDH, HCG, CEA, AFP - CT neck, chest, abdomen - Biopsy lymph node | Non-Seminomatous Germ Cell Tumor | IV |
| 74M | 1. Plasma Cell 2. Liver/Bile Duct | - Blood tests: SPEP, serum free light chains - Bone marrow biopsy - PET CT - MRI cholangiopancreas - Galleri re-test: Negative | Smoldering Myeloma | High-Risk |
| 59M | 1. Prostate 2. Lymphoid | - Blood tests: PSA, CBC, flow cytometry, LDH - PET CT - Biopsy lymph node - Biopsy skin | Cutaneous T Cell Lymphoma | IV |

***** Out-of-state patients that were seen in the DFCI MCED clinic for initial evaluation but were referred to local collaborating oncologist for completion of work-up.

F, Female; M, Male; CT, Computed Tomography; PET, Positron Emission Tomography; CEA, Carcinoembryonic Antigen; CA19-9, Carbohydrate Antigen 19-9; AFP, Alpha Fetoprotein; MRI, Magnetic Resonance Imaging; PSA, Prostate Specific Antigen; LDH, Lactate Dehydrogenase; HCG, Human Chorionic Gonadotropin; SPEP, Serum Protein Electrophoresis; CBC, Complete Blood Count
