# Supplementary material for: Diagnostic Outcomes among Patients with Positive Multi-Cancer Early Detection Test Results
Source: Cancer Res Commun. 2026 Mar 9;6(3):511–5. doi: 10.1158/2767-9764.CRC-25-0723 (PMC13012066; doi:10.1158/2767-9764.CRC-25-0723)
Supplement: Supplementary Table 2 — shows our general approach for performing a clinical evaluation of a patient with a cancer signal-detected test result. [file crc-25-0723_supplementary_table_2_suppst2.docx]

**Supplementary Table 2.** Recommendations for Diagnostic Approach by Cancer Signal Origin

| **Cancer Signal Origin** | **Diagnostic Tests** |
| --- | --- |
| Anus | 1. Direct exam, Pap smear, HPV testing 2. High resolution anoscopy 3. Biopsy |
| Bladder, Urothelial tract | 1. Urinalysis 2. CT abdomen/pelvis or CT urogram 3. Cystoscopy, If CT unrevealing 4. IR- or cystoscopy-guided biopsy |
| Bone and Soft tissue | 1. CT chest, abdomen, pelvis or PET-CT scan 2. Biopsy |
| Breast | 1. Physical exam, Diagnostic mammogram 2. Breast ultrasound (if abnormality detected on exam) and/or Breast MRI 3. Breast biopsy |
| Cervix | 1. Direct exam, Pap smear, HPV testing 2. Colposcopy with biopsy |
| Colon, Rectum | 1. CEA 2. Colonoscopy with biopsy 3. If colonoscopy unrevealing, consider CT abdomen/pelvis, capsule study, and PET scan |
| Head and Neck | 1. Direct exam, Nasopharyngoscopy 2. CT head and neck 3. Consider PET scan 4. Biopsy |
| Hematopoietic and Lymphoid Organs | Lymphoid lineage:   1. CBC with differential, LDH, flow cytometry 2. PET scan 3. Excisional lymph node biopsy   Myeloid lineage:   1. CBC with differential, flow cytometry 2. Bone marrow biopsy   Plasma cell lineage:   1. CBC with differential, complete chemistry panel, serum protein electrophoresis, serum free light chains 2. Bone marrow biopsy 3. Complete skeletal imaging – whole body MRI v PET-CT scan |
| Kidney | 1. Urinalysis 2. MRI kidneys with contrast or triphasic renal CT 3. Biopsy |
| Liver, Bile duct | 1. AFP, CA19-9, CEA 2. Abdominal ultrasound 3. Multiphasic CT liver or MRI cholangiopancreas (MRCP) 4. ERCP/EUS or IR-guided biopsy |
| Lung | 1. CT Chest 2. PET scan if CT detects abnormality 3. IR or bronchoscopy/EBUS biopsy |
| Melanocyte-containing tissues/skin | 1. Physical exam 2. Biopsy |
| Ovary | 1. CA-125 2. Transvaginal ultrasound 3. CT abdomen/pelvis with contrast or MRI 4. Biopsy |
| Pancreas, Gallbladder | 1. CA19-9, CEA, CA-125 2. Multiphase contrast-enhanced CT or MRI with MRCP 3. EUS-guided biopsy or ERCP |
| Prostate | 1. PSA 2. MRI Prostate 3. Biopsy |
| Stomach, Esophagus | 1. Upper endoscopy 2. Biopsy |
| Thyroid | 1. Physical exam 2. Thyroid ultrasound 3. Biopsy |
| Uterus | 1. Transvaginal ultrasound 2. Endometrial biopsy |

HPV, Human Papillomavirus; CT, Computed Tomography; IR, Interventional Radiology; PET, Positron Emission Tomography; MRI, Magnetic Resonance Imaging; CEA, Carcinoembryonic Antigen; CBC, Complete Blood Count; AFP, Alpha Fetoprotein; CA19-9, Carbohydrate Antigen 19-9; ERCP, Endoscopic Retrograde Cholangiopancreatography; MRCP, Magnetic Resonance Cholangiopancreatography; EBUS, Endobronchial Ultrasound; CA-125, Cancer Antigen 125; EUS, Endoscopic Ultrasound; PSA, Prostate Specific Antigen; LDH, Lactate Dehydrogenase; HCG, Human Chorionic Gonadotropin; SPEP, Serum Protein Electrophoresis
